# Mapping Proximity to Infectious Disease Physicians Across the United States

**DOI:** 10.1093/ofid/ofae208

**Published:** 2024-04-18

**Authors:** Julian Maamari, Zhuo Chen, Issam Motairek, Sadeer Al-Kindi, Jorge Fleisher

**Affiliations:** Department of Internal Medicine, St. Elizabeth's Medical Center—A Boston University Teaching Hospital, Brighton, Massachusetts, USA; Division of Public Health, Infectious Diseases and Occupational Medicine, Department of Medicine, Mayo Clinic, Rochester, Minnesota, USA; Harrington Heart and Vascular Institute, University Hospitals, and School of Medicine, Case Western Reserve University, Cleveland, Ohio, USA; Department of Internal Medicine, Cleveland Clinic, Cleveland, Ohio, USA; Department of Cardiology, DeBakey Heart & Vascular Institute, Houston Methodist Hospital, Houston, Texas, USA; Department of Internal Medicine, St. Elizabeth's Medical Center—A Boston University Teaching Hospital, Brighton, Massachusetts, USA; Division of Infectious Diseases, St. Elizabeth's Medical Center—A Boston University Teaching Hospital, Brighton, Massachusetts, USA

**Keywords:** geographic access, health equity, NRMP match, physician shortage, rural infectious disease

## Abstract

Enduring shortages of infectious disease physicians across the United States continue despite efforts to mitigate the problem. The recent fellowship match results underscore the difficulty in rectifying that shortage. Our report sheds light on the current geographic distribution of US infectious disease physicians and highlights the challenges faced by rural communities.

Infectious disease (ID) physicians have a central role in both inpatient and outpatient care. However, the seemingly insurmountable shortage of ID physicians continues to present a barrier to patients requiring that care. Despite intensified efforts by the Infectious Diseases Society of America (IDSA) to recruit more physicians and the briefly lived increased interest in ID during the pandemic, the number of ID fellowship applicants continues to lag behind the number of available positions. In the 2024 match cycle, only 50.8% of programs were able to fill all their positions, with only 303 or 67.3% of positions filled. This represents a decrease in the absolute number of positions filled from 328 positions the year prior and comes at a time when a staggering 79.5% of counties in the United States do not have a single ID physician [1].

To better understand the current status of the ID workforce and reflect upon recent changes since the recent COVID-19 pandemic, we analyzed the geographic distribution and distance of patients from ID physicians. In September 2023, we obtained data on national provider identification from the Centers for Medicare and Medicaid National Plan and Provider Enumeration System (NPI). We then filtered the data set for Infectious Disease physicians, defined according to infectious disease taxonomy number 207RI0200X. Physicians were then geocoded by longitude and latitude using their primary business addresses. The distance between each physician's location and the centroid of each US Census block was calculated. The national median distance to the nearest ID physician was determined, weighted by census block population (based on 2020 census), and stratified by urban vs rural areas (Census Bureau classification).

As previously described [2], our analyses were conducted using QGIS, version 3.22, Python, version 3.9.12, and Geopandas, version 0.11.0. Institutional review board approval was not required as no patient-level data were used. Of 8132968 census blocks in the United States, 4191709 of the census blocks belong to urban areas with a population of ∼265 million, while 3941259 of the census blocks belong to rural areas with a population of ∼66 million individuals.

A total of 12230 ID physicians were identified. We presumed that all physicians within the database are currently active. The overall median distance to an ID physician (interquartile range [IQR]) was 6.98 km (4.33 [3.21–17.98] km), with that number increasing to 30.50 km (18.95 [16.66–54.90] km) for those living in rural areas (Figure 1). Only 32.05% of individuals in rural America are <20 km away from their nearest provider compared with 88.2% in urban counties. Furthermore, though one cannot derive any conclusion from visual comparison, and though many confounders come into play, the map appears to correlate with similar heat maps such as those of vaccine hesitancy and overall life expectancy [3]. The average ratio of ID physicians was 3.6/100000 individuals, representing a slight improvement from prior years [4]. Comparatively, however, there are close to 4 times as many cardiologists across the United States. In addition, a visual comparison of a cardiologist distribution heat map shows both a wider distribution of cardiologists in the rural West and Midwestern United States, as well as a wider distribution in states with key areas lacking providers such as New York, Michigan, and the more central states [2]. Similarly, primary care facilities are also more widely distributed across the country, as seen in data compiled by the US Health Resources and Services Administration (HRSA). A 2021 European survey found that the ratio of ID physicians ranged from <0.5/100000 individuals to 7.8/100000 individuals in Sweden [5]. The lack of a recommended ratio, confounded by differences in medical systems and trained providers, makes it difficult to assess the optimal ratio. Each country would need to take a closer look at its ID workforce and their allocations into different sectors—including health care, public policy, private companies, etc.—to determine the optimal ratio of physicians. The HRSA projects the need for 15700 ID physicians by 2025, amounting to a recommended ratio of roughly 4.64/100000 individuals [6].

**Figure 1.**
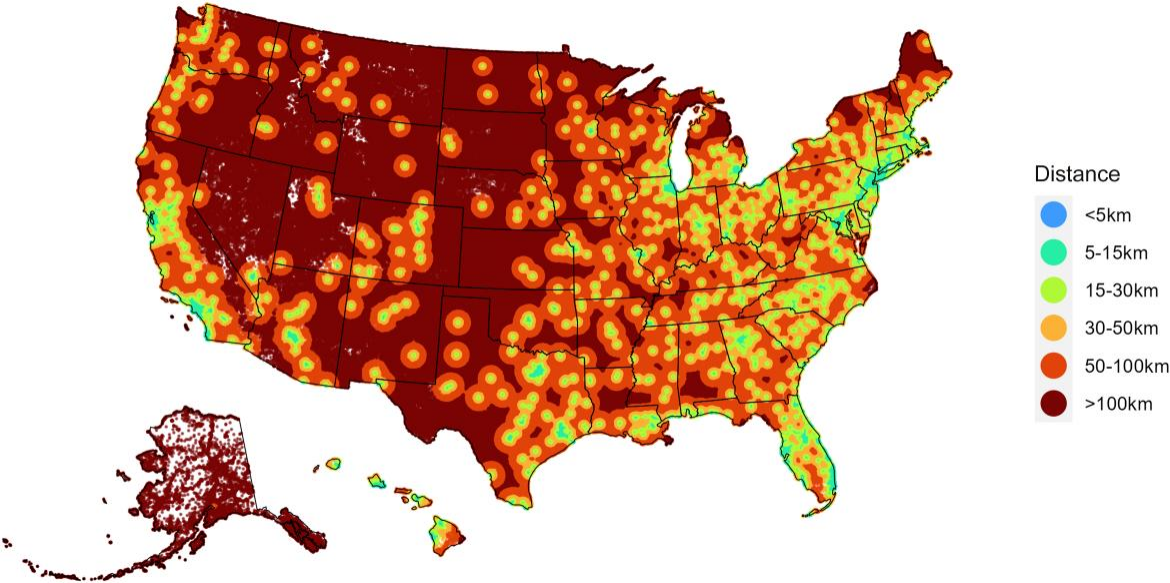
Map of mean distance from each census block to an infectious disease physician.

Overall, these numbers are a stark reflection of the persistent scarcity of ID physicians within the workforce, albeit with a slight improvement over the past decade. From a patient perspective, access to care may be limited by both proximity and socioeconomic factors. People with HIV or other chronic infections, members of the LGBTQI+ community, intravenous drug users, and individuals requiring regular injections are among those in particular need of access to specialized care and services. The issue of proximity can be partially tackled by telehealth services that providers in more urban settings often provide. Though these services may certainly be a bridge to solving the issue of proximity, some conditions often require an in-person evaluation, and many providers prefer initial evaluations to be in-person. Therefore, much remains to be done to strengthen access to ID physicians and the critical services they provide. Financial incentives, visa and immigration facilitations, early mentorship, and improved working conditions are some of the pursued efforts that may bolster recruitment within ID and expand their availability across different areas.

Some limitations to the presented data must be mentioned. For one, ID physicians are not the sole providers of specialized ID care in the United States, with nurse practitioners and physician assistants picking up larger roles in some settings. The NPI database itself also carries some inaccuracies. For example, some ID trained physicians may take up roles in government, hospital epidemiology and infection control, or primary care and hospitalist medicine, thus allotting less time to ID-focused care. Overall, we believe these figures to be largely reflective of the current status, the trend that is shaping ID in the United States, and the work that remains to be done.
